# The effect of eugenol anesthesia on the electric organ discharge of the weakly electric fish *Apteronotus leptorhynchus*

**DOI:** 10.1007/s10695-023-01259-5

**Published:** 2023-11-24

**Authors:** Dávid Lehotzky, Annika I. Eske, Günther K. H. Zupanc

**Affiliations:** Laboratory of Neurobiology, Department of Biology, Northeastern University, Boston, MA 02115 USA

**Keywords:** Anesthesia, *Apteronotus leptorhynchus*, Electric organ discharge, Eugenol, Neuro-behavioral assay, Pacemaker nucleus

## Abstract

Eugenol, the major active ingredient of clove oil, is widely used for anesthesia in fish. Yet virtually nothing is known about its effects on CNS functions, and thus about potential interference with neurophysiological experimentation. To address this issue, we employed a neuro-behavioral assay recently developed for testing of water-soluble anesthetic agents. The unique feature of this *in-vivo* tool is that it utilizes a readily accessible behavior, the electric organ discharge (EOD), as a proxy of the neural activity generated by a brainstem oscillator, the pacemaker nucleus, in the weakly electric fish *Apteronotus leptorhynchus*. A deep state of anesthesia, as assessed by the cessation of locomotor activity, was induced within less than 3 min at concentrations of 30–60 µL/L eugenol. This change in locomotor activity was paralleled by a dose-dependent, pronounced decrease in EOD frequency. After removal of the fish from the anesthetic solution, the frequency returned to baseline levels within 30 min. Eugenol also led to a significant increase in the rate of ‘chirps,’ specific amplitude/frequency modulations of the EOD, during the 30 min after the fish’s exposure to the anesthetic. At 60 µL/L, eugenol induced a collapse of the EOD amplitude after about 3.5 min in half of the fish tested. The results of our study indicate strong effects of eugenol on CNS functions. We hypothesize that these effects are mediated by the established pharmacological activity of eugenol to block the generation of action potentials and to reduce the excitability of neurons; as well as to potentiate GABA_A_-receptor responses.

## Introduction

Over the last few decades, legal requirements for minimizing pain, suffering, or distress inflicted upon protected animals during experiments have prompted the search for suitable anesthetics and the optimization of methods used for anesthesia. Ideal anesthetics are cheap and readily available; easy to administer and have minimal toxic effects on the animal, the handler, and the environment; provide a wide margin of safety; induce anesthesia rapidly and lead to quick recovery after removal of the animal from the anesthetic. Moreover, if the research project involves examination of nervous function, the effect of the anesthetic on the neural system under study should be minimal.

For anesthesia in fish, no currently available drug meets all the above requirements, but several agents satisfy some of them. For each of the common drugs, doses have been established that induce and/or maintain anesthesia in selected fish taxa (Neiffer and Stamper 2009). However, a significant gap in knowledge exists about possible neural effects of these anesthetics. To address this issue, we recently developed a neuro-behavioral assay (Eske et al. 2023). The unique feature of this *in-vivo* tool is that a well-defined and readily accessible behavior serves as a proxy of the pattern of the neural command signals that drive the frequency of this behavior.

The behavior utilized by the assay is the electric organ discharge (EOD) of the weakly electric fish *Apteronotus leptorhynchus*. These discharges are generated continuously at frequencies of approximately 650–1,000 Hz, with males occupying a frequency band between 700 Hz and 1,000 Hz, and females discharging within a frequency band ranging from 650 to 800 Hz, at a water temperature of 26 °C (Zupanc et al. 2014). The EOD is generated by an electric organ composed of axonal terminals (so-called electrocytes) of modified spinal motoneurons (so-called electromotoneurons) (de Oliveira-Castro 1955; Bennett 1971; Waxman et al. 1972). The synchronized discharges of individual electrocytes constitute the EOD.

The frequency of the EOD is controlled by the frequency of the oscillations of a central pattern generator in the medulla oblongata, the pacemaker nucleus (Pn) (for review see Dye and Meyer 1986). Its neural output drives the EOD in a one-to-one fashion, i.e., the frequency of the EOD equals the frequency of the synchronized oscillations of the Pn (Meyer 1984). Thus, the frequency of the EOD can be used as a proxy of the frequency of the Pn oscillations.

In fish similar in size to the ones used in the present study, the Pn consists of roughly 200,000 cells, of which approximately 5,000 are neurons (Sîrbulescu et al. 2014). However, neuroanatomical and neurophysiological evidence, as well as simulations based on computational modeling, suggest that a relatively small neural network of approximately 90 pacemaker cells and 20 relay cells is sufficient to produce the sustained high-frequency oscillations of the Pn (Elekes and Szabo 1985; Dye and Heiligenberg 1987; Dye 1991; Heiligenberg et al. 1996; Moortgat et al. 2000; Sîrbulescu et al. 2014; Zupanc et al. 2014, 2019; Hartman et al. 2021; Ilieş and Zupanc 2023). The pacemaker cells are connected via chemical and electrotonic synapses with each other and the relay cells. The latter project to the spinal electromotoneurons.

Modulations of the EOD involve changes in frequency and/or amplitude. Some of them are limited to a few milliseconds. The most common type of these transient modulations is chirps, which consist of specific frequency increases and amplitude decreases (Zupanc and Maler 1993; Engler et al. 2000; Engler and Zupanc 2001; Zupanc et al. 2006). At the other extreme are modulations that become manifest in the course of weeks, such as frequency decreases experimentally induced by β-estradiol (Meyer et al. 1987; Schaefer and Zakon 1996; Zupanc et al. 2014); they mimic the development of the sexual dimorphism in EOD frequency in *A. leptorhynchus*.

Neurophysiological experiments and simulations based on computational modeling have suggested two major types of neural mechanisms underlying EOD modulations. One type operates within the Pn. For example, changes in potassium equilibrium potential caused by alterations in the capacity of astrocytes to buffer extracellular K^+^ have been proposed to mediate changes in EOD frequency (for review see Zupanc 2020). A second type is based on input received by the Pn from other brain regions (for review see Metzner 1999). One of these inputs is potentially involved in mediating the effect of eugenol on the EOD, as will be discussed in detail below (see Discussion, ‘Molecular and cellular mechanisms of the anesthetic effect of eugenol on the EOD frequency in *A. leptorhynchus*: a working hypothesis’). This excitatory input is received by the relay cells from the mesencephalic sublemniscal prepacemaker nucleus (SPPn) via NMDA receptors. The SPPn, in turn, is under tonic inhibition from a subnucleus of the nucleus electrosensorius in the diencephalon, the nE↓. The latter input utilizes GABA as a transmitter and activates GABA_A_ receptors.

In the present study, we have employed the neuro-behavioral assay to examine the effect of eugenol on the EOD of *A. leptorhynchus*. This phenylpropanoid is the major active component of clove oil; other active ingredients are isoeugenol and methyl-eugenol. Clove oil and each of its three active ingredients have been widely used as fish anesthetics. Yet, only very recently possible effects of eugenol on physiological functions in the central nervous system (CNS) have attracted attention by investigators (Machnik et al. 2023). The results of our investigation indicate a pronounced, dose-dependent effect of eugenol on the frequency of the neural oscillations of the Pn, commencing within less than a minute after the fish’s exposure to the anesthetic and lasting for up to 30 min after the fish’s removal from the immersion solution. These findings call for caution when conducting neurophysiological experiments on fish under general anesthesia with eugenol.

## Materials and methods

### Animals

A total of 8 adult (approximately 2-year-old) *A. leptorhynchus*, collected in their natural habitat in Colombia and obtained through a tropical fish importer (Segrest Farms, Gibsonton, Florida, USA), were used in this study. Their total lengths (body weights) ranged from 122 mm to 157 mm (4.0 g to 6.9 g), with medians of 135 mm and 6.1 g, respectively. The baseline EOD frequencies of the fish indicated that this sample contained both males (high frequencies) and females (low frequencies) (cf. Zupanc et al. 2014).

The fish were kept isolated in their home tanks (50 cm × 30 cm × 30 cm) under a 12:12-h light:dark photoperiod at water temperatures of 26–29 °C, conductivities of 180–310 µS/cm, and pH values of 7.7–7.9. Each tank was equipped with an opaque cylindrical tube (length: 190 mm; inner diameter: 38 mm; outer diameter: 42 mm), which provided shelter for the fish. During the experiments, the water temperature was sampled every 10 min with a calibrated digital thermometer (Fisher Brand, Model 15–077-8, 11.705,843; Thermo Fisher Scientific, Waltham, Massachusetts, USA; accuracy ± 0.05 °C).

### EOD recording

**Fig. 1:**
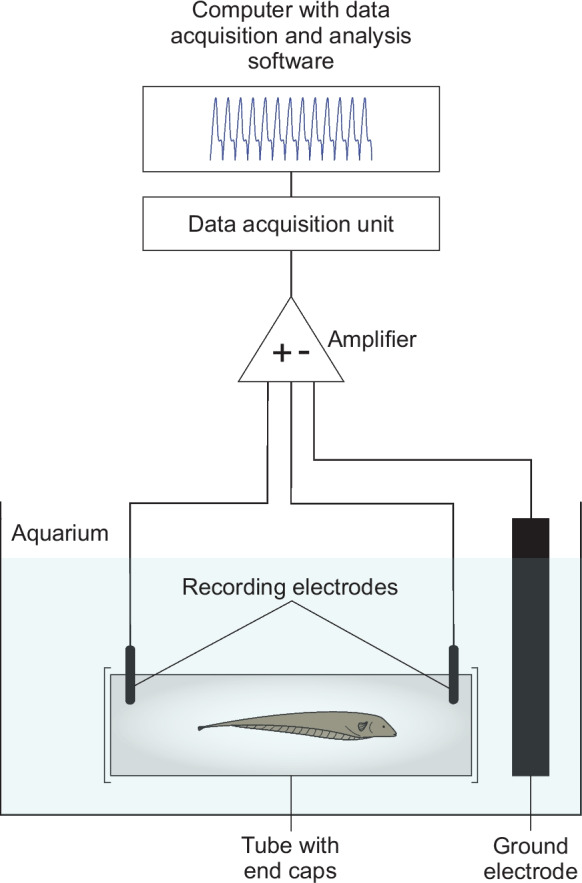
Experimental setup for recording of the fish’s EOD (from Eske et al. 2023)

Differential recording of the fish’s EOD was performed through a pair of stainless-steel electrodes built into the shelter tube (Fig. 1). During the experiment, the open ends of the tube were closed with a course plastic mesh netting to ensure that the fish remained close to the recording electrodes, thereby yielding high-quality recordings. This restraint is minimally invasive, as, particularly during daytime, the fish remain in shelter tubes with open ends for prolonged periods of time, often for several hours. The recorded EOD signal was amplified and digitized following a protocol established previously (Eske et al. 2023).

### Calculation of EOD frequency

For calculation of EOD frequency, the method described by Eske et al. (2023) was implemented using MATLAB version R2021b with the following modifications: The time series data were filtered in a moving time window using a bandpass filter with frequency band $\left[0.4, 1.4\right]\times {f}_{d, k}$, where ${f}_{d,k}$ is the dominant frequency of window $k$ inside the frequency range $\left[0.9, 1.1\right]\times {f}_{d, k-1}$ determined by the previous window’s dominant frequency ${f}_{d, k-1}$. For each window $k=1, 2,\dots$, the dominant frequency ${f}_{d,k}$ was computed based on the power spectrum of the time series data using fast Fourier transform and the “findpeaks” function of MATLAB. The initial dominant frequency ${f}_{d,0}$ was determined as the dominant frequency of the baseline recording.

### Temperature adjustment

All frequencies were adjusted to a reference temperature of 26 °C, using a Q_10_ of 1.56 (Zupanc et al. 2003).

### Coefficient of variation

The variability of sampled EOD frequency measurements was characterized by computing the coefficient of variation (*cv*), defined as

1 $$cv = \frac{standard\;deviation}{mean} 100 (\%)$$

### Chirp detection

**Fig. 2:**
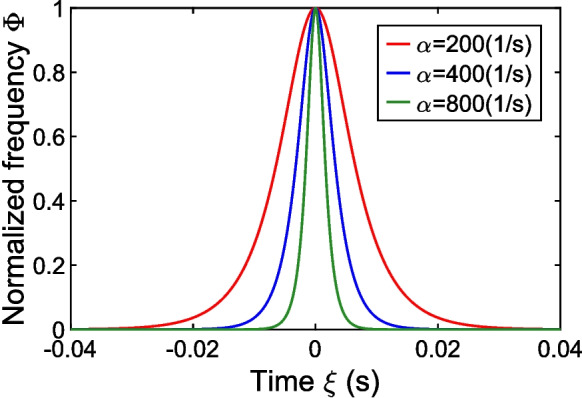
Frequency shape of chirps normalized by peak frequency rise used for chirp detection as described in (Eske et al. 2023). During chirping, the time course of normalized frequency was modeled by a single-parameter function $\Phi \left(\upxi ;\mathrm{\alpha }\right)$ (see Eq. 2). Time is denoted by $\upxi$ and different colors correspond to different shape parameter values α

We employed the method described in Eske et al. (2023) for the detection of chirps. This method assumes that the frequency shape of chirps normalized by the peak frequency rise is described in time by function

2 $$\Phi \left(\upxi ;\mathrm{\alpha }\right)=\frac{2{e}^{\mathrm{\alpha \xi }}}{1+{e}^{2\mathrm{\alpha \xi }}}$$

where $\upxi$ is the time coordinate measured from the chirp’s peak frequency and $\mathrm{\alpha }$ is an unknown parameter identified by the chirp detection method (Fig. 2). This function closely matches the shape of type-2 chirps, and, though to a lesser extent, the shape of type-1 chirps. Since the present study investigates the effect of anesthesia on type-2 chirps, only chirps with maximum frequency rises of less than 150 Hz were included in our analysis, which is a defining property of type-2 chirps. By contrast, type-1 chirps exhibit maximum frequency rises between 200 and 350 Hz (Engler et al. 2000).

### Model fitting of EOD frequency recovery

The EOD frequency of each recording was normalized with respect to its baseline frequency. For each eugenol concentration, the following model was fitted on the resulting normalized frequency data points after the fish’s exposure to the anesthetic solution:

3 $$F\left(t\right)=a+\frac{\left(c-a\right)t+d{t}^{2}}{b+t}$$

Here $t=0$ corresponds to the time instant when the fish was returned to its home tank. The fitting of model parameters $a, b, c,$ and $d$ was carried out according to the method described in (Eske et al. 2023).

### Statistical analysis

Statistical analysis of the data was performed using R version 4.2.3. Throughout this paper, significance levels were set at $p<0.01$ (2-tailed).

To assess differences in the change of EOD frequency and chirp rate after anesthesia, we employed the Related-Samples Sign Test using the “rstatix” package (Kassambara 2023) in R. To minimize the number of fish involved in our experiments, we chose the smallest number of fish $n$ required for significance under the expected test statistic (0 or $n$): $n=\lceil1-{\text{lo}}{\text{g}}_{2}\left(0.01\right)\rceil=8.$

To assess dose-dependent differences in recovery, we used Split-Plot ANOVA based on the following linear mixed effects model:

4 $${Y}_{ijk}={\delta }_{0}+{\delta }_{1}{c}_{i}+{\beta }_{j\left(i\right)}+{\delta }_{2}{t}_{k}+{\delta }_{12}{c}_{i}{t}_{k}+{\varepsilon }_{ijk}$$

Here, ${Y}_{ijk}$ denotes the measured normalized frequency data after anesthesia, with the indices being associated with eugenol treatment $i\in \left\{0,\dots ,3\right\}$ ($i=0$ corresponds to control), fish $j\in \left\{1,\dots ,8\right\}$, and time instance $k\in \left\{1,\dots ,360\right\}$. Parameter ${\updelta }_{0}$ is the overall mean, ${\delta }_{1}$ denotes the effect of eugenol concentration ${c}_{i}$, ${\beta }_{j\left(i\right)}\sim \mathcal{N}\left(0,{\sigma }_{\beta }^{2}\right)$ is the random effect of fish $j$ under treatment $i$, ${\updelta }_{2}$ is the effect of time ${t}_{k}=k/2$ (min), ${\updelta }_{12}$ is the concentration-dependent effect of time, and ${\varepsilon }_{ijk}\sim \mathcal{N}\left(0,{\sigma }_{\varepsilon }^{2}\right)$ is the measurement error (${\sigma }_{\beta }^{2}$ and ${\sigma }_{\varepsilon }^{2}$ are unknown variances).

This model assumes that, for a given treatment, the recovery curve of normalized frequency is independent of the fish. To fit this model on the measured data and to assess significance, we used the “lmerTest” package (Kuznetsova et al. 2017) in R.

To determine the time duration of recovery at each eugenol concentration ($i>0$), we fitted for time windows $q=0, 1, \dots , 345$ Eq. 4 on the normalized frequency data ${Y}_{ijk}$ associated with the given eugenol concentration and control, and with the time window $k\in \left\{q+1,\dots ,q+15\right\}$. For each $r=1, 2, \dots$, we tested the null hypothesis that in time windows $q=1, \dots , r,$ ${\updelta }_{1}=0$ and ${\updelta }_{12}=0$ both hold. We applied the Bonferroni correction to assess significance and assigned the recovery time ${t}_{r}$ to the first $r$ value where the null hypothesis was not rejected.

### Experimental design

**Fig. 3:**
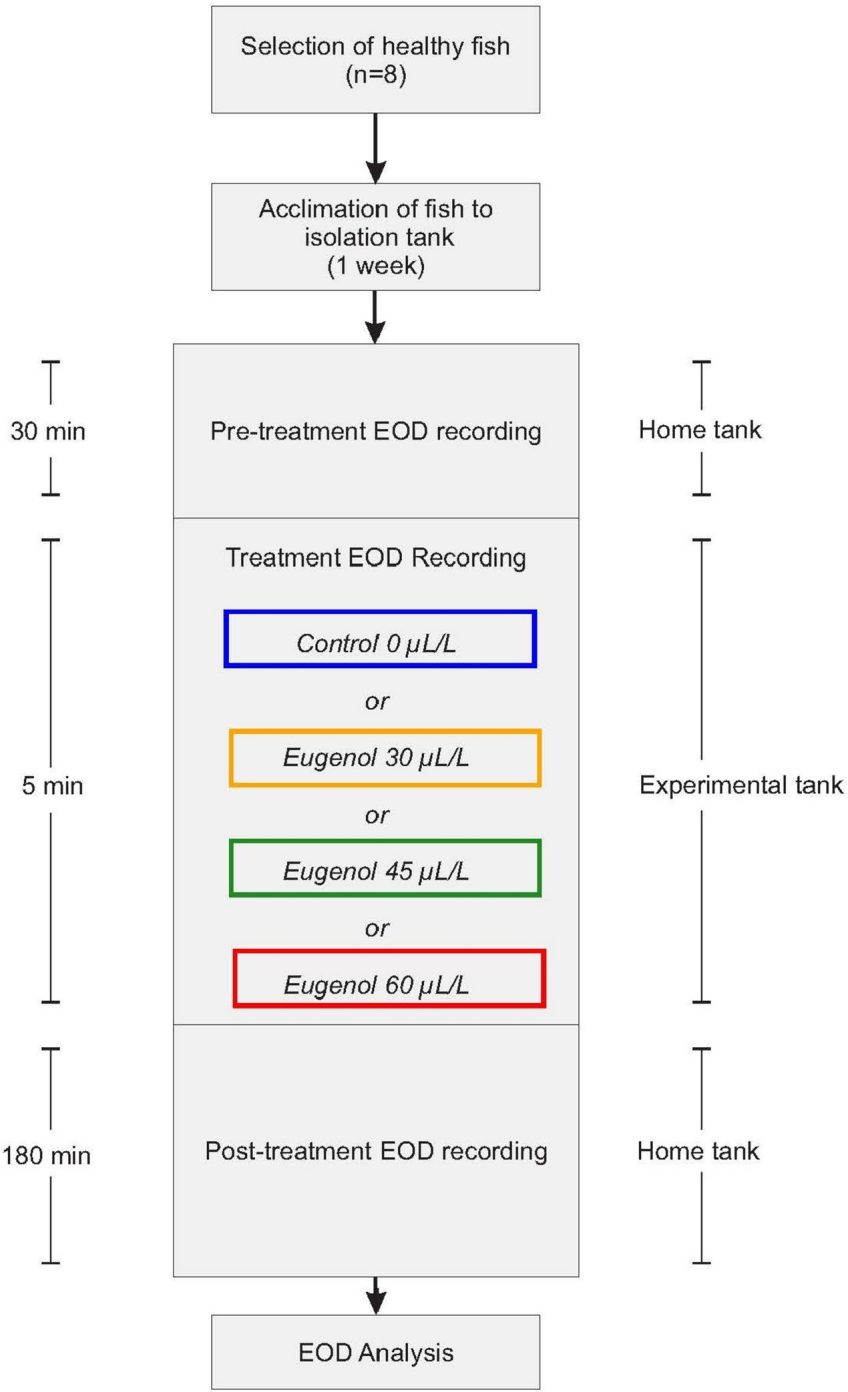
Flow diagram outlining the experimental design

To examine the effect of eugenol on EOD frequency and chirping behavior, the following experiments were conducted using 8 fish (Fig. 3): First, the baseline EOD was recorded in the fish’s home tank (50 cm × 30 cm × 30 cm) for 30 min. Then, for the 5-min treatment session, the shelter tube with the fish was transferred to a smaller (experimental) tank (30 cm × 20 cm × 20 cm) containing either eugenol dissolved in aquarium water at a certain concentration (30 µL/L, 45 µL/L, or 60 µL/L; ‘test condition’) or aquarium water only (‘control condition’). The temperature in the experimental tank was similar to the temperature in the home tank (median difference: ± 0.2 °C). Finally, the shelter tube with the fish was returned to the home tank, where recording continued for 180 min. Throughout the 215 min of pre-treatment, treatment, and post-treatment, the recording of the EOD was interrupted only for a few seconds during which the fish was transferred between its home tank and the experimental tank. The order of the four treatment conditions was determined using a randomized complete block design. Each test or control experiment in a given fish was conducted on a different day.

## Results

### Effect of eugenol anesthesia on locomotor activity

Most of the time, fish in the shelter tube maintained an upright position, with their longitudinal axis parallel to the walls of the tube. Forward and backward swimming occurred frequently and appeared to be mediated primarily by high-frequency undulations of the anal fin. Sporadically, the fish made 180° turns around its vertical axis.

Upon exposure of the fish to eugenol, body movements, including undulation of the anal fin, slowed down. When these movements ceased, the fish lost equilibrium and lay on one side. Quantitative analysis of the locomotor activity in 8 fish revealed that the time after which body movements ceased was dose dependent: 1–3 min (mean: 1.8 min) at 30 µL/L eugenol, 1–2 min (mean: 1.4 min) at 45 µL/L eugenol, and 1 min in each fish at 60 µL/L eugenol.

Whereas the time after which the movement of the body stopped at a given eugenol concentration was rather robust, the time after which the anal fin ceased undulating was quite variable. At a concentration of 30 µL/L, in 3 out of 8 fish, the anal fin continued undulating throughout the 5-min exposure of the fish to eugenol. In the other 5 fish, undulation stopped after 1–3 min. At a concentration of 45 µL/L, in 1 out of 8 fish the anal fin continued to undulate throughout the 5-min eugenol anesthesia. In the other 7 fish, the anal fin ceased undulating after 1–3 min. At a concentration of 60 µL/L, the anal fin stopped undulating in each of the 8 fish during anesthesia; this effect required eugenol exposure times ranging from 1 to 4 min.

### Effect of eugenol anesthesia on EOD frequency

**Fig. 4:**
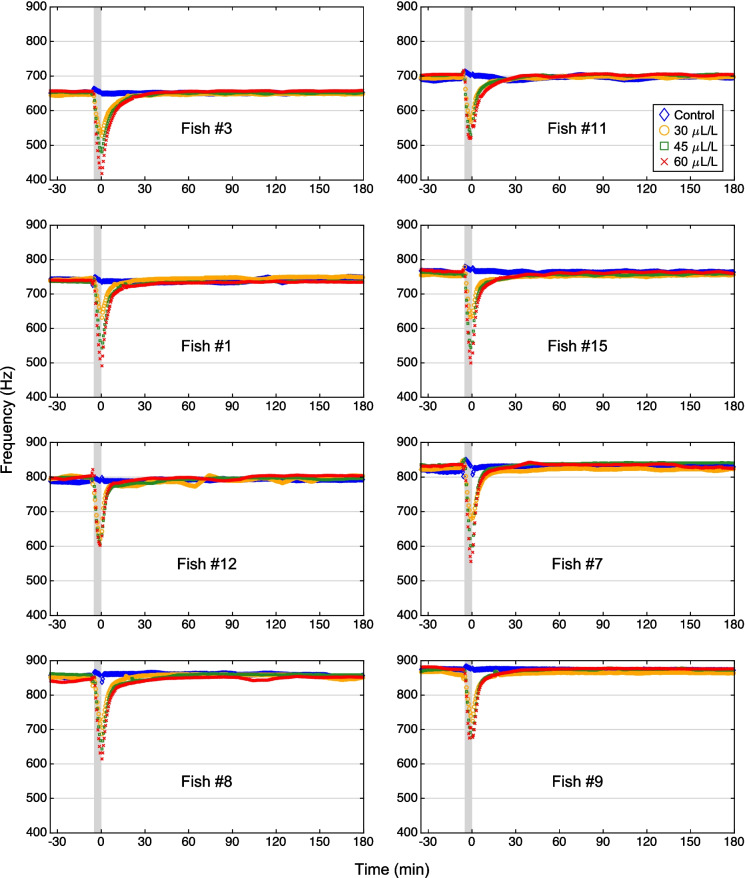
Effect of eugenol anesthesia on EOD frequency. The frequency was determined at 30-s intervals through a pair of electrodes built into a shelter tube. To establish the baseline frequency, the EOD was recorded in the fish’s home tank for 30 min. Then, the shelter tube with the fish was transferred to a smaller tank containing aquarium water from the home tank (control: *blue diamonds*) or eugenol in aquarium water at one of three concentrations (30 µL/L: *orange circles*; 45 µL/L: *green squares*; 60 µL/L: *red crosses*). After 5 min (indicated by the *gray bar*), the fish was returned to its home tank, where recording of the EOD continued for another 180 min. The return of the fish to its home tank was arbitrarily defined as time point ‘0’. Each of the 4 experiments was conducted on a different day. Note that the baseline EODs of the 8 individual fish cover a wide range of frequencies, including those typical of females (low frequencies) and males (high frequencies). The plots of the individual fish are presented in ascending order according to their baseline EOD frequency

For determining the baseline EOD frequency of each of the 8 fish, the 30-min recordings of their EODs before each treatment (exposure to water from the home tank only or to eugenol at one of the three concentrations) were used. Then, the median frequencies at 30-s intervals within 1-min time windows of each of these 30-min recordings were computed, yielding a total of 59 median frequency values. As expected, based on our selection criterion, the frequencies of individual fish differed substantially (Fig. 4). However, the EOD frequencies within each of the 32 pre-treatment time periods of 30 min were highly stable, as indicated by the small *cv* values ranging from 0.12% to 0.59%.

**Fig. 5:**
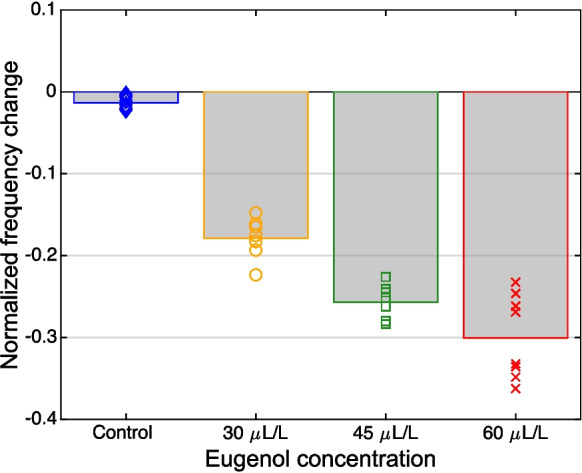
Dose-dependent effect of eugenol on EOD frequency. The plot is based on the EOD recordings shown in Fig. 4. For the analysis, the minimum value of median EOD frequencies determined at 30-s intervals was related to the median EOD frequency determined during the 30 min immediately preceding the 5 min of the fish’s exposure to the anesthetic solution. For the control experiment (*blue*) and each of the 3 eugenol concentrations (30 µl/L: *orange*; 45 µl/L: *green*; 60 µl/L: *red*), the normalized maximum frequency drops exhibited by each of the 8 fish are shown (*diamonds*, *circles*, *squares*, and *crosses*, respectively), together with the medians (*bars*) of these values

In each of the 8 fish examined, eugenol anesthesia resulted, within minutes, in a pronounced decrease in EOD frequency (Fig. 4). The magnitude of decrease was dose dependent (Figs. 4 and 5). The largest effect was observed at the highest concentration of eugenol (60 µL/L), which induced maximum frequency drops of 184–277 Hz (median: 232 Hz), compared to the corresponding median baseline frequency determined during the 30 min immediately preceding the anesthesia.

Comparison of the medians of the EOD frequency during the 30 min before the start of the anesthesia, and during the 15 min after the onset of anesthesia (i.e., including the 5 min of the fish’s exposure to the eugenol solution and the first 10 min after their return to the respective home tank) revealed a significant difference, in response to eugenol, whereas the corresponding control experiments had no significant effect (Related Samples Sign Test, *n* = 8 fish; for details see Table 1).

**Table 1:** Difference in median EOD frequency change between the 30-min interval before and the 15-min interval after the start of anesthesia. Significant differences are indicated by *

	Control	30 µL/L	45 µL/L	60 µL/L
*p*-value	0.2891	0.0078*	0.0078*	0.0078*
Median (Hz)	1.16	-48.18	-65.54	-88.65
Range (Hz)	[-4.68, 12.15]	[-65.83, -33.57]	[-92.80, -58.62]	[-121.68, -52.50]

**Fig. 6:**
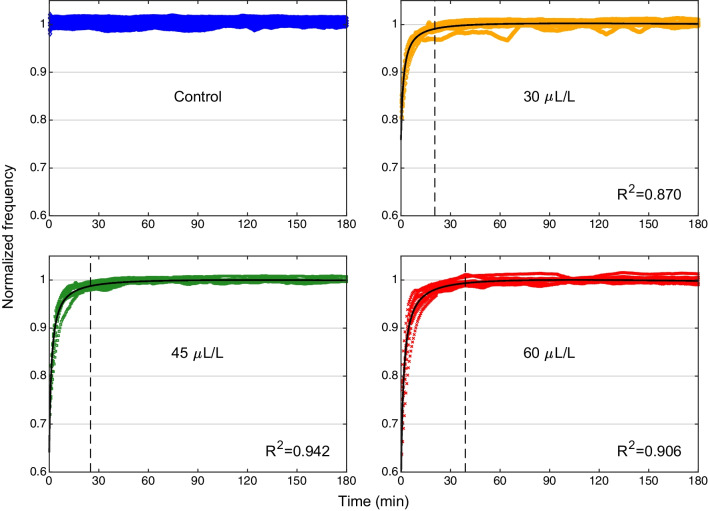
Recovery of EOD frequency after eugenol anesthesia. The graph is based on the EOD recordings shown in Fig. 4. The recovery is characterized by the EOD frequencies normalized to the median frequency of the 30-min EOD recording prior to the fish’s transfer to the small tank with water from its home tank (control: *blue diamonds*) or one of the three concentrations of eugenol in aquarium water (30 µl/L: *orange circles*; 45 µl/L: *green squares*; 60 µl/L: *red crosses*). The return of the fish to its home tank is arbitrarily defined as time point ‘0’. Data points for all 8 fish are shown together with the 4-parameter model fitted using nonlinear regression (*black curves*). The fitted model parameters are summarized in Table 2. The time point of recovery is indicated with vertical *black dashed lines*

After transfer of the anesthetized fish back to their home tanks, the EOD frequency returned to baseline levels within approximately 30 min (Fig. 6). The time course of recovery followed the model described by Eq. 3 (see *black curves* in Fig. 6; see Table 2 for fitted model parameters). Comparisons of the maximum decrease in normalized frequency (with respect to baseline EOD frequency) revealed significant differences between different eugenol concentrations (*p* = 0.008, Related Samples Sign Test, *n* = 8 fish; for details see Fig. 6 and Table 3).

**Table 2:** Parameters identified for the model described by Eq. 3**,** using nonlinear regression on normalized frequency data points associated with the recovery part of eugenol anesthesia (cf. legend of Fig. 6)

Parameter (unit)	30 µL/L	45 µL/L	60 µL/L
$a$(1)	0.7465	0.6344	0.5649
$b$(min)	1.4568	1.4064	1.5816
$c$(1)	1.0089	1.0084	1.0131
$d$(min^−1^)	-3.2299 $\times$ 10^–5^	-3.3821 $\times$ 10^–5^	-6.2321 $\times$ 10^–5^

**Table 3:** Difference in the largest normalized EOD frequency drops induced by different treatment protocols

	30 µL/L vs. Control	45 µL/L vs. 30 µL/L	60 µL/L vs. 45 µL/L
*p*-value	0.0078*	0.0078*	0.0078*
Median	-0.1650	-0.0715	-0.0349
Range	[-0.2202, -0.1262]	[-0.1231, -0.0169]	[-0.1000, -0.0056]

### Effect of eugenol anesthesia on chirp production rate

**Fig. 7:**
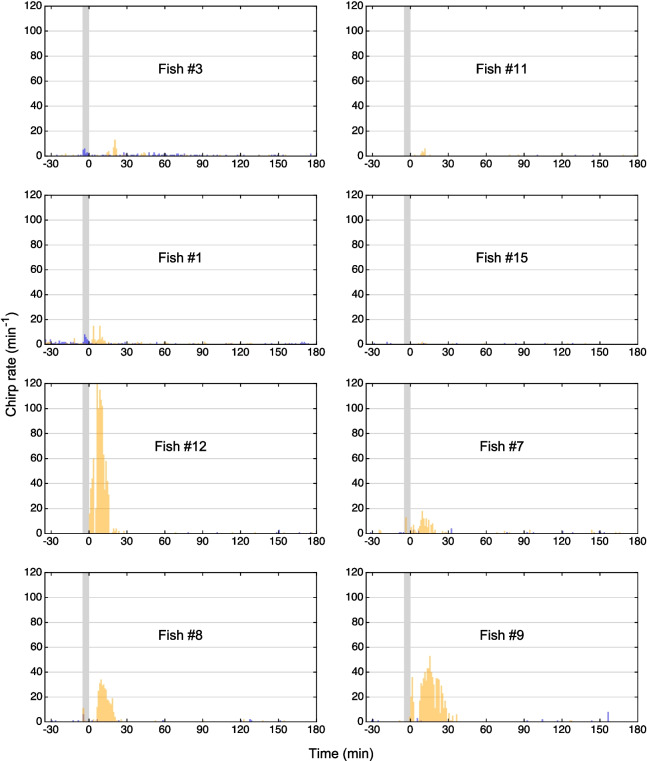
Effect of eugenol anesthesia at a concentration of 30 µL/L on the generation of type-2 chirps. Chirp detection was based on the same EOD recordings that were used for the time–frequency plots shown in Fig. 4. Eugenol anesthesia for 5 min (indicated by the *gray bar*) induced an increase in the rate of chirping (*orange bars*), particularly during the 30 min following the fish’s exposure to eugenol, compared to the chirp rates prior to anesthesia or during the control experiments (*blue bars*). Note inter-individual differences in the effect of eugenol on the number of chirps produced

**Fig. 8:**
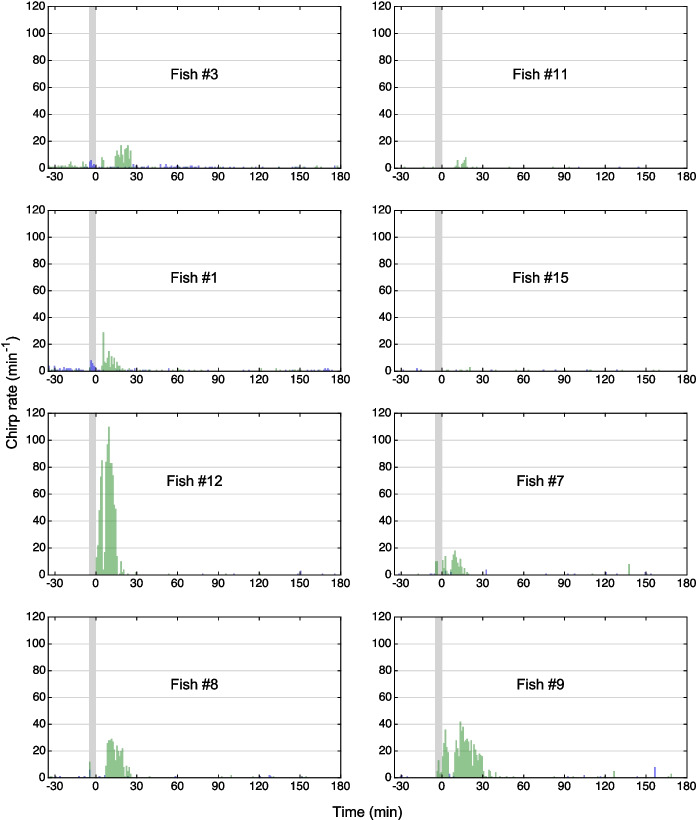
Effect of eugenol anesthesia at a concentration of 45 µL/L on the generation of type-2 chirps. Eugenol anesthesia for 5 min (indicated by the *gray bar*) induced an increase in the rate of chirping (*green bars*), particularly during the 30 min following the fish’s exposure to eugenol, compared to the chirp rate prior to anesthesia or the control experiments (*blue bars*)

**Fig. 9:**
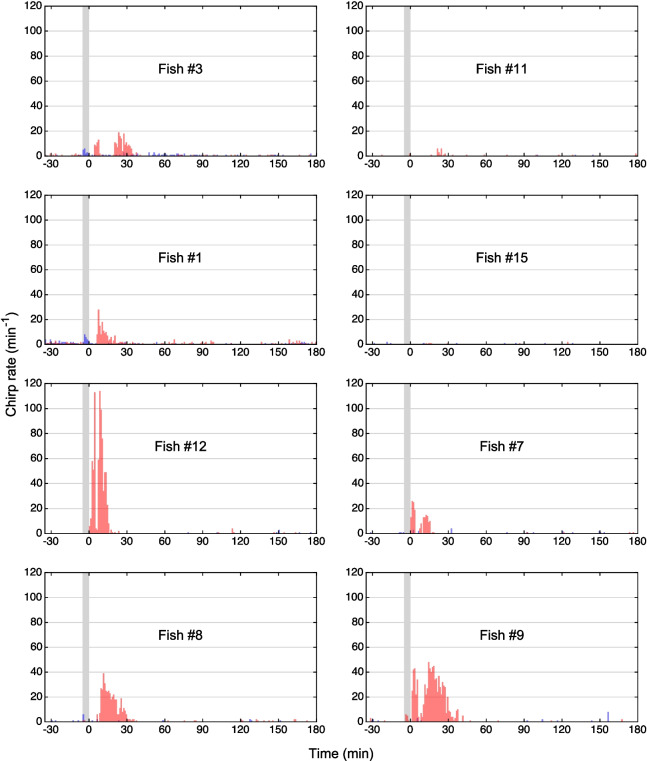
Effect of eugenol anesthesia at a concentration of 60 µL/L on the generation of type-2 chirps. Eugenol anesthesia for 5 min (indicated by the *gray bar*) induced an increase in the rate of chirping (*red bars*), compared to pre-anesthesia and control levels (*blue bars*)

Chirp rates were very low prior to anesthesia at any of three eugenol concentrations or to the control treatment (range: 0–1.57 chirps/min; median: 0.05 chirps/min; *n* = 32 pre-treatment EOD recordings over 30 min each, based on 4 pre-treatment recordings in each of the 8 fish) (Figs. 7, 8 and 9).

**Fig. 10:**
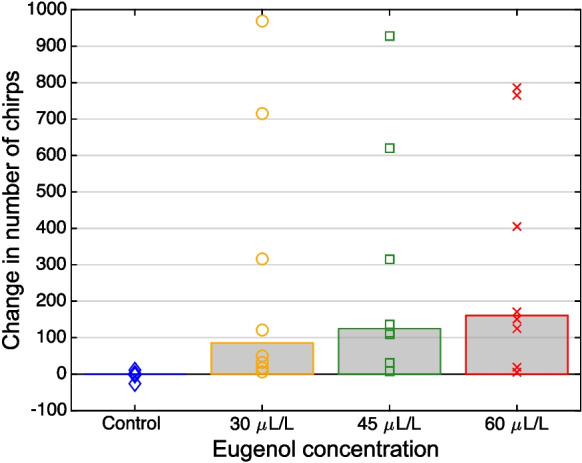
Effect of eugenol anesthesia on the number of type-2 chirps. The change in the number of type-2 chirps between the 30 min interval preceding the fish’s exposure to the anesthetic and the 30 min interval after its exposure was determined for the control experiment (*blue*) and each of the 3 eugenol concentrations (30 µl/L: *orange*; 45 µl/L: *green*; 60 µl/L: *red*) for each of the 8 fish (*diamonds*, *circles*, *squares*, and *crosses*, respectively). For the control and the three eugenol concentrations, the median values are indicated with *bars*

In each of the 8 fish, eugenol induced an increase in the number of chirps produced. This effect was largely restricted to the 30 min immediately following the 5-min exposure of the fish to the anesthetic. During this period, at each of the three eugenol concentrations tested, the increase in the number of chirps compared to the 30 min before anesthesia was significantly higher than for the control treatment (*p* = 0.008, Related-Samples Sign Test, *n* = 8 fish) (Figs. 7, 8, 9 and 10, Table 4). However, the magnitude of the increase in chirp rate was independent of the concentration of eugenol used (Figs. 7, 8, 9 and 10; Table 5).

**Table 4:** Difference in increase in the number of chirps induced by each of the three eugenol concentrations versus control. Changes in chirp rate were compared between the 30-min interval before anesthesia and the 30-min interval after exposure to the anesthetic

	30 µL/L vs. Control	45 µL/L vs. Control	60 µL/L vs. Control
*p*-value	0.0078*	0.0078*	0.0078*
Median	97.5	137	160
Range	[9, 969]	[11, 928]	[8, 786]

**Table 5:** Difference in increase in the number of chirps induced by anesthesia between pairs of different eugenol concentrations. Changes in chirp rate were compared between the 30-min interval before anesthesia and the 30-min interval after exposure to the anesthetic

	45 µL/L vs. 30 µL/L	60 µL/L vs. 45 µL/L	60 µL/L vs. 30 µL/L
*p*-value	0.7266	0.7266	0.2891
Median	8	23	60.5
Range	[-95, 77]	[-163, 166]	[-204, 119]

### Effect of eugenol anesthesia on EOD amplitude

**Fig. 11:**
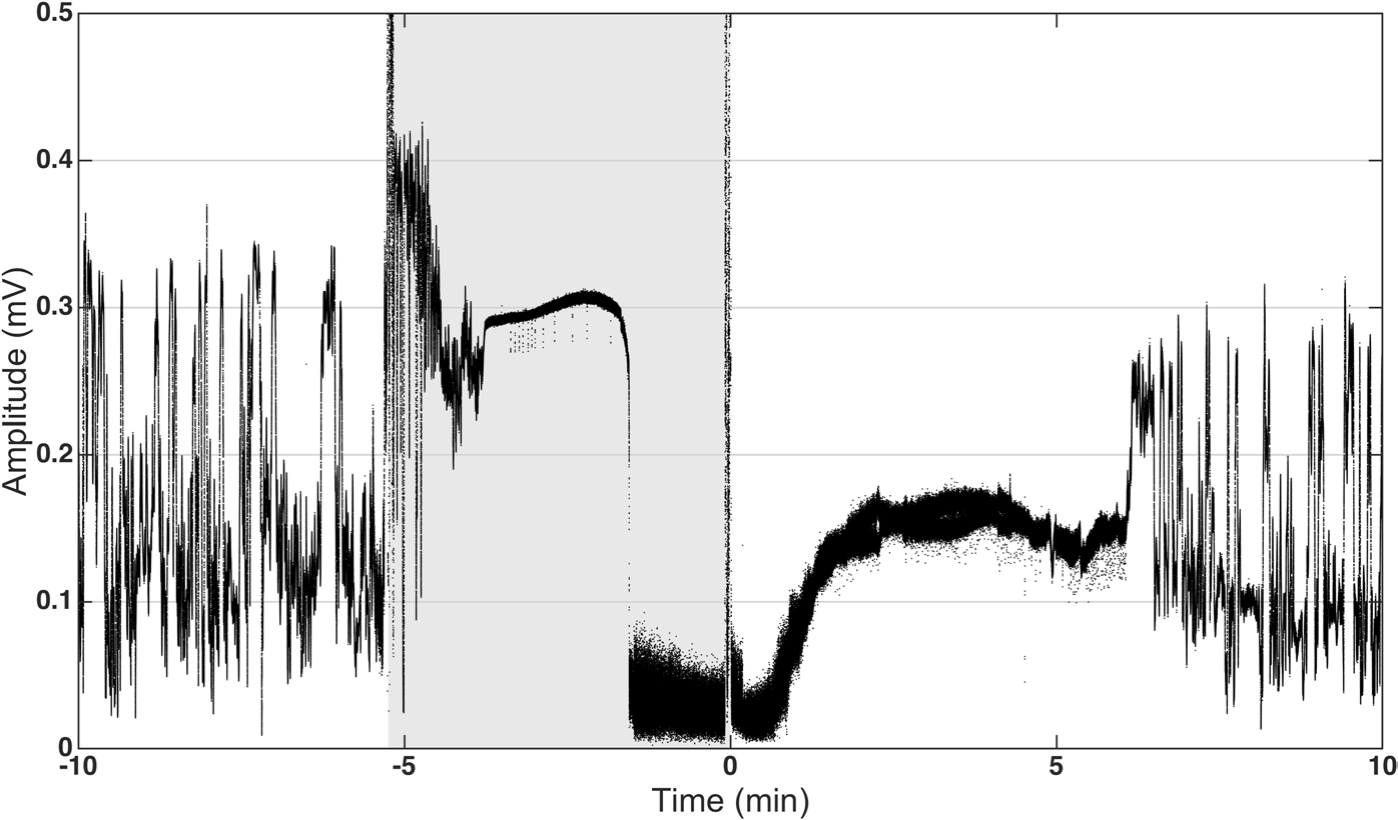
Collapse of EOD amplitude during high-concentration-eugenol anesthesia. The recording was taken during exposure of Fish #9 to a eugenol concentration of 60 µL/L (cf. Figure 4). The peak-to-peak amplitude of the EOD waveform is plotted as a function of time. The start and the end of the 5-min anesthesia (*gray area*) are indicated by the two amplitude surges. Amplitude jitter was caused by the fish’s movements relative to the recording electrodes fixed to the inside of the shelter tube. Approximately 1.5 min after the onset of the anesthesia, the fish’s movements ceased, and, thus, the jittering of the EOD amplitude became minimal. Approximately 3.5 min after the start of the anesthesia, the EOD amplitude collapsed to very low levels. Within 1 min of return of the fish to its home tank, the EOD amplitude gradually recovered. It reached baseline levels approximately 6 min after the end of the fish’s exposure to the eugenol solution

In 4 out of 8 fish exposed to 60 µL/L eugenol, the EOD amplitude collapsed to markedly lower levels approximately 3.5 min after the onset of anesthesia (Fig. 11). During the remaining time of the fish’s exposure to the anesthetic, the amplitude either remained at this low level (in 1 fish) or even decreased (in 3 fish). After transferring the fish back to its home tank, the EOD amplitude gradually returned to baseline levels within a few minutes.

## Discussion

### Eugenol as a fish anesthetic

Clove oil and its active ingredients have been tested as sedatives and anesthetics in numerous fish species (for review see Priborsky and Velisek 2018). Although their use in fish intended for human consumption is not approved in some countries (such as the United States; FDA 2007), or is conditional upon compliance with maximum residue limits in foodstuffs in other countries (such as the member states of the European Union; European Commission 2011), these compounds have become increasingly common over the last few decades for anesthesia of ornamental fish and fish used in research.

In the present study, we found that, in *A. leptorhynchus,* concentrations of 45 µL/L and 60 µl/L eugenol induce anesthesia characterized by total loss of equilibrium and cessation of any movement, including undulation of the anal fin, within 1–4 min. This result is comparable to observations made in two other small-sized tropical/subtropical freshwater fish, zebrafish (*Danio rerio*) and red garra (*Garra rufa*). Both species belong to the same teleostean superorder (Ostariophysi) as *A. leptorhynchus*. In *D. rerio*, a stage of anesthesia similar to the one induced in *A. leptorhynchus* was achieved with a concentration of 60 µL/L eugenol within 1.5 min (Grush et al. 2004). In *G. rufa*, total loss of equilibrium and movement, as well as absence of reaction to external stimuli, was observed at eugenol concentrations of 25 µL/L and 50 µL/L after approximately 3 min and 1.5 min, respectively (Aydın 2022).

In the latter investigation, it was furthermore shown that induction time significantly decreases with stepwise increases in eugenol concentrations from 12.5 µL/L to 150 µL/L. This effect is reminiscent of our finding in *A. leptorhynchus* of a dose-dependent decrease in time at which locomotor activity ceases.

### Effects of eugenol on CNS function

#### High efficacy in crossing the blood–brain barrier

At the eugenol concentrations used in the present study (30–60 µL/L), the fish started to lower the EOD frequency 1–2 min after their transfer into the anesthetic solution. This very short latency is comparable to the latencies observed in a previous investigation when the fish were anesthetized with MS-222 or urethane (Eske et al. 2023). However, the latter two anesthetics required significantly higher concentrations for inducing frequency decreases after similar latencies — 200 mg/L in the case of MS-222 and 25 g/L in the case of urethane. Since a critical step toward exerting the frequency-lowering effects in the Pn is the ability of anesthetics to cross the blood–brain barrier, it appears reasonable to assume that the efficacy of eugenol in this step is higher than those of MS-222 and urethane. As is well established in clinical practice, almost all drugs that interfere with brain function are lipid soluble and small, with a molecular mass < 400 Da (Pardridge 2012). Each of the three anesthetics used in our two studies meet the latter criterion (molecular masses: eugenol, 164 g/mol; MS-222, 261 g/mol; urethane, 89 g/mol). Unfortunately, the octanol–water partition coefficients are available only for eugenol (log k_ow_ = 2.49) (National Center for Biotechnology Information 2023a) and urethane (log k_ow_ = -0.15) (National Center for Biotechnology Information 2023b). However, these data support our hypothesis that, while the solubility of eugenol in water (2460 mg/L at 25 °C; Yalkowsky et al. 2010) is sufficient for dissolving the compound at the concentrations used in the present study, its lipophilicity enables the molecule to cross the blood–brain barrier through lipid-mediated transport rapidly and with high efficacy.

#### Comparative aspects of the effect of eugenol on CNS function

Despite the wide use of eugenol and related compounds as anesthetics in fish, little is known about their effects on CNS function. A first attempt to fill this critical gap in knowledge was undertaken recently by employing an antidromic-stimulation regime of the axon of the Mauthner cell to study the effect of different concentrations of isoeugenol on the generation and propagation of action potentials in goldfish (*Carassius auratus*) (Machnik et al. 2023). No effect was observed at 10 mg/L and 20 mg/L, even after exposure of the fish to the anesthetic for more than 1 h. At 40 mg/L isoeugenol, a slight decrease in the slope of action potentials was evident after 10–30 min. Only at the highest concentration tested, 60 mg/L, and exposure of the fish for at least 10–30 min, additional aspects of the action potential were affected, including peak amplitude and duration. However, even the latter changes were rather modest, compared to controls. By contrast, sensory stimulation by brief acoustic or visual pulses had more pronounced effects on postsynaptic potentials induced in the Mauthner neuron, with visual stimuli evoking stronger responses than acoustic stimuli. The authors interpreted these results as evidence that isoeugenol acts primarily locally on sensory systems, instead of affecting action potential generation and propagation, as well as chemical and electric transmission, at the level of central neurons. Nonetheless, the limitation of their study to just one neuron, and the lack of experiments examining possible effects of isoeugenol on synaptic transmission, hamper such generalization.

In contrast to this study, the findings of the present investigation demonstrate that eugenol can induce strong dose-dependent effects on the function of a brain system, even at low concentration and within a few minutes. These observations are consistent with the results of experiments carried out on rat neocortical and hippocampal slices that demonstrated reversible and dose-dependent suppression by eugenol of epileptiform field potentials and spreading depression, presumably via inhibition of synaptic plasticity (Müller et al. 2006). On the other hand, it is important to keep in mind that direct comparison of our study and that of Machnik et al. (2023) is not possible because not only were different fish species (*A. leptorhynchus vs. C. auratus*) and different neural systems (pacemaker nucleus *vs.* Mauthner neuron) examined but also different, though closely related, agents (eugenol *vs.* isoeugenol) were employed.

#### Fast recovery of EOD frequency after eugenol anesthesia

Assaying the EOD frequency as a proxy of the output frequency of the Pn enabled us not only to analyze, with high temporal resolution, the drop in frequency induced by eugenol, but also to track the recovery of the EOD frequency after the fish’s return from the anesthetic solution to the water of its home tank. Although the fish regained equilibrium and locomotor activity, including undulation of the anal fin, within a few minutes (data not shown), the EOD frequency remained reduced significantly longer, for up to 30 min. Both the drop in EOD frequency during anesthesia and the persistence of this effect beyond the immediate state of anesthesia underscore the need to consider the possibility of effects on CNS function whenever neurophysiological experiments on fish are performed under anesthesia with eugenol (or related compounds).

Compared to fish anesthetics tested previously by employing the neuro-behavioral assay (Eske et al. 2023), it is notable that the recovery time after eugenol anesthesia is markedly shorter than the recovery time of urethane (approximately 3 h) and MS-222 (approximately 1 h). Thus, the relatively short ‘neural’ recovery period of eugenol is another attractive feature of this anesthetic, in addition to the advantages mentioned in section ‘Eugenol as a fish anesthetic,’ above.

### Molecular and cellular mechanisms of the anesthetic effect of eugenol on the EOD in *A. leptorhynchus*: a working hypothesis

#### A mechanistic model for explaining the effect of eugenol on EOD frequency but not on chirping behavior

In the following, we relate what is known about the pharmacological actions of eugenol to the neural circuitry involved in generating and modulating the output frequency of the pacemaker nucleus, thereby providing a mechanistic model of the effect of this anesthetic on the EOD frequency. We abstain from presenting a similar model for explaining the effect of eugenol on chirping behavior. The generation of chirps by the pacemaker nucleus is primarily controlled by a projection to its relay cells, originating in a subnucleus of the central posterior/prepacemaker nucleus, the so-called CP/PPn-C. This control is exerted by glutamatergic synaptic transmission and activation of non-NMDA-type receptors. The CP/PPn-C, in turn, receives a multitude of inputs from other brain regions (for reviews see Metzner 1999; Zupanc 2002). However, unlike the connection between the CP/PPn-C and the relay cells, the nature (excitatory, inhibitory) of these inputs to the CP/PPn-C, and the transmitters/receptors involved at their synapses, are unknown. We, therefore, can only speculate that one or more of these inputs are targets of eugenol, and it is this action that results in the increase in chirping.

#### Eugenol limits the generation of action potentials

Several cellular and molecular effects of eugenol have been implicated in the anesthetic activity of eugenol. Notably, its ability to block the generation of nerve impulses and to reduce the excitability of neurons (Kozam 1977; Trowbridge et al. 1982; Brodin and Røed 1984; Moreira-Lobo et al. 2010) has been postulated to underlie its property as a local and general anesthetic (for review see Chung and Oh 2013). Possible molecular targets of this action are voltage-gated sodium channels, which are inhibited by eugenol (Park et al. 2009; Wang et al. 2015). In Na_v_ 1.7-transfected CHO cells, this molecule preferentially binds to such channels in the inactivated state, thereby driving fast-inactivated channels into slow-inactivated channels (Wang et al. 2015). In addition, methyl-eugenol has been shown to exert inhibitory effects on Kv1.5 and, possibly, other potassium channels (Li et al. 2007). The resulting inhibition of K^+^ currents may lead to elongation of the duration of action potentials and the refractory period.

In the Pn of *A. leptorhynchus*, the inhibitory effects of eugenol on voltage-gated sodium channels and voltage-gated potassium channels may limit the generation of action potentials and result in the prolongation of action-potentials, respectively. We propose that the combination of these effects reduces the frequency of the sustained high-frequency oscillations of the Pn. This hypothesis is congruent with experimental evidence and predictions obtained through modeling, which have suggested the extracellular potassium concentration and the potassium channel conductances as critical determinants of the frequency of the spontaneous oscillations of this brainstem nucleus (Zupanc et al. 2014, 2019; Hartman et al. 2021). The notion that the effect of anesthetics, including MS-222 and urethane (Eske et al. 2023), as well as eugenol (present study), is mediated by the action of these anesthetics directly in the Pn, but not by modulation of afferent input, receives strong support by electrophysiological recording from isolated Pn tissue (G.K.H. Zupanc, unpublished observations).

#### Eugenol potentiates the GABA_A_-receptor response

A second mechanisms that may contribute to the effect of eugenol to induce a drop in EOD frequency is its ability to potentiate GABA_A_-receptor response. Such an effect has been observed in test systems based on GABA_A_ receptors expressed in *Xenopus* oocytes (Aoshima and Hamamoto 1999; Sahin et al. 2017)^1^ and is consistent with the results obtained in a [^3^H] muscimol-binding assay using rat cortical homogenate (Kheawfu et al. 2022). Since GABA application to the SPPn lowers the frequency of the EOD, and thus the output frequency of the Pn (Metzner 1999), a potentiation of the response of GABA receptors received by input from the nE↓ would be expected to result in a decrease of the oscillation frequency of the Pn.

A similar hypothesis related to the potentiation of the inhibitory function of GABA_A_ receptors has been suggested as a mechanistic explanation for the drop in EOD frequency caused by urethane but not MS-222 (Eske et al. 2023). Remarkably, the time courses of the frequency change during the fish’s exposure to eugenol, as shown in the present study, and urethane resemble each other strikingly but both differ significantly from such a plot obtained after MS-222 anesthesia. We hypothesize that such differences in the ‘signatures’ of the time–frequency plots established through the neuro-behavioral assay reflect differences in the cellular and molecular mechanisms mediating the reduction in EOD frequency.

## Data Availability

Data that support the findings of this study are available from the corresponding author upon reasonable request.

## Footnotes

1 A different effect of eugenol was observed in a study that examined how eugenol affects GABA-induced current in rat trigeminal ganglia and in human embryonic kidney 293 cells expressing the GABA_A_ receptor subtype α1β2γ2 (Lee SH, Moon JY, Jung SJ, Kang JG, Choi SP, Jang JH (2015) Eugenol inhibits the GABA_A_ current in trigeminal ganglion neurons. PLoS One 10:e0117316. https://doi.org/10.1371/journal.pone.0117316). The authors found that it inhibits GABA_A_ current in a reversible, dose-dependent manner. The reason for the discrepancy between the studies of Aoshima and Hamamoto (1999), Sahin et al. (2017), and Kheawfu et al. 2022) on the one hand, and of Lee et al. (2015) on the other, is unclear.

## References

[CR1] Aoshima H, Hamamoto K (1999). Potentiation of GABA_A_ receptors expressed in *Xenopus* oocytes by perfume and phytoncid. Biosci Biotechnol Biochem.

[CR2] Aydın B (2022). Anaesthetic efficacy of eugenol in doctor fish (*Garra rufa*): behavioural and cardiovascular responses. Aquac Res.

[CR3] Bennett MVL (1971) Electric organs. In: Hoar WS, Randall DJ (eds) Fish Physiology, Vol 5: Sensory Systems and Electric Organs Academic Press, New York, pp 347–491

[CR4] Brodin P, Røed A (1984). Effects of eugenol on rat phrenic nerve and phrenic nerve-diaphragm preparations. Arch Oral Biol.

[CR5] Chung G, Oh SB, Ramawat KG, Mérillon J-M (2013). Eugenol as local anesthetic. Natural products: phytochemistry, botany and metabolism of alkaloids, phenolics and terpenes.

[CR6] de Oliveira-Castro G (1955). Differentiated nervous fibers that constitute the electric organ of *Sternarchus albifrons*. Linn an Acad Bras Cienc.

[CR7] Dye J (1991). Ionic and synaptic mechanisms underlying a brainstem oscillator: an in vitro study of the pacemaker nucleus of *Apteronotus*. J Comp Physiol A.

[CR8] Dye J, Heiligenberg W (1987). Intracellular recording in the medullary pacemaker nucleus of the weakly electric fish, *Apteronotus*, during modulatory behaviors. J Comp Physiol A.

[CR9] Dye JC, Meyer JH, Bullock TH, Heiligenberg W (1986). Central control of the electric organ discharge in weakly electric fish. Electroreception.

[CR10] Elekes K, Szabo T (1985). Synaptology of the medullary command (pacemaker) nucleus of the weakly electric fish (*Apteronotus leptorhynchus*) with particular reference to comparative aspects. Exp Brain Res.

[CR11] Engler G, Zupanc GKH (2001). Differential production of chirping behavior evoked by electrical stimulation of the weakly electric fish, *Apteronotus leptorhynchus*. J Comp Physiol A.

[CR12] Engler G, Fogarty CM, Banks JR, Zupanc GKH (2000). Spontaneous modulations of the electric organ discharge in the weakly electric fish, *Apteronotus leptorhynchus*: a biophysical and behavioral analysis. J Comp Physiol A.

[CR13] Eske AI, Lehotzky D, Ahmed M, Zupanc GKH (2023). The effect of urethane and MS-222 anesthesia on the electric organ discharge of the weakly electric fish *Apteronotus leptorhynchus*. J Comp Physiol A.

[CR14] European Commission (2011) Commission Regulation (EU) No 363/2011 of 13 April 2011 amending the Annex to Regulation (EU) No 37/2010 on pharmacologically active substances and their classification regarding maximum residue limits in foodstuffs of animal origin, as regards the substance isoeugenol. European Commission, Brussels

[CR15] FDA (2007) Concerns related to the use of clove oil as an anesthetic for fish. CVM GFI #150. U.S. Department of Health and Human Services, Food and Drug Administration, Center for Veterinary Medicine, Rockville, MD

[CR16] Grush J, Noakes DLG, Moccia RD (2004). The efficacy of clove oil as an anesthetic for the zebrafish, *Danio rerio* (Hamilton). Zebrafish.

[CR17] Hartman D, Lehotzky D, Ilieş I, Levi M, Zupanc GKH (2021). Modeling of sustained spontaneous network oscillations of a sexually dimorphic brainstem nucleus: the role of potassium equilibrium potential. J Comput Neurosci.

[CR18] Heiligenberg W, Metzner W, Wong CJH, Keller CH (1996). Motor control of the jamming avoidance response of *Apteronotus leptorhynchus*: evolutionary changes of a behavior and its neuronal substrates. J Comp Physiol A.

[CR19] Ilieş I, Zupanc GKH (2023). Computational modeling predicts regulation of central pattern generator oscillations by size and density of the underlying heterogenous network. J Comput Neurosci.

[CR20] Kassambara A (2023) Pipe-friendly framework for basic statistical test. R package version 0.7.2. Available from https://rpkgs.datanovia.com/rstatix/. Accessed on 25 April 2023

[CR21] Kheawfu K, Pikulkaew S, Wellendorph P, LvG J, Rades T, Müllertz A, Okonogi S (2022). Elucidating pathway and anesthetic mechanism of action of clove oil nanoformulations in fish. Pharmaceutics.

[CR22] Kozam G (1977). The effect of eugenol on nerve transmission. Oral Surg Oral Med Oral Pathol.

[CR23] Kuznetsova A, Brockhoff PB, Christensen RHB (2017). lmerTest Package: tests in linear mixed effects models. J Stat Softw.

[CR24] Lee SH, Moon JY, Jung SJ, Kang JG, Choi SP, Jang JH (2015). Eugenol inhibits the GABA_A_ current in trigeminal ganglion neurons. PLoS One.

[CR25] Li HY, Park C-K, Jung SJ, Choi S-Y, Lee SJ, Park K, Kim JS, Oh SB (2007). Eugenol inhibits K^+^ currents in trigeminal ganglion neurons. J Dent Res.

[CR26] Machnik P, Biazar N, Schuster S (2023). Recordings in an integrating central neuron reveal the mode of action of isoeugenol. Commun Biol.

[CR27] Metzner W (1999). Neural circuitry for communication and jamming avoidance in gymnotiform electric fish. J Exp Biol.

[CR28] Meyer JH (1984). Steroid influences upon discharge frequencies of intact and isolated pacemakers of weakly electric fish. J Comp Physiol A.

[CR29] Meyer JH, Leong M, Keller CH (1987). Hormone-induced and maturational changes in electric organ discharges and electroreceptor tuning in the weakly electric fish *Apteronotus*. J Comp Physiol A.

[CR30] Moortgat KT, Bullock TH, Sejnowski TJ (2000). Gap junction effects on precision and frequency of a model pacemaker network. J Neurophysiol.

[CR31] Moreira-Lobo DCA, Linhares-Siqueira ED, Cruz GMP, Cruz JS, Carvalho-de-Souza JL, Lahlou S, Coelho-de-Souza AN, Barbosa R, Magalhães PJC, Leal-Cardoso JH (2010). Eugenol modifies the excitability of rat sciatic nerve and superior cervical ganglion neurons. Neurosci Lett.

[CR32] Müller M, Pape H-C, Speckmann E-J, Gorji A (2006). Effect of eugenol on spreading depression and epileptiform discharges in rat neocortical and hippocampal tissues. Neuroscience.

[CR33] National Center for Biotechnology Information (2023a) PubChem Compound Summary for CID 3314, Eugenol. Available from https://pubchem.ncbi.nlm.nih.gov/compound/Eugenol. Accessed on September 27, 2023

[CR34] National Center for Biotechnology Information (2023b) PubChem Compound Summary for CID 5641, Urethane. Available from https://pubchem.ncbi.nlm.nih.gov/compound/Urethane. Accessed on September 27, 2023

[CR35] Neiffer DL, Stamper MA (2009). Fish sedation, analgesia, anesthesia, and euthanasia: considerations, methods, and types of drugs. ILAR J.

[CR36] Pardridge WM (2012). Drug Transport across the Blood-Brain Barrier. J Cereb Blood Flow Metab.

[CR37] Park C-K, Kim K, Jung SJ, Kim MJ, Ahn DK, Hong S-D, Kim JS, Oh SB (2009). Molecular mechanism for local anesthetic action of eugenol in the rat trigeminal system. Pain.

[CR38] Priborsky J, Velisek J (2018). A review of three commonly used fish anesthetics. Rev Fish Sci Aquac.

[CR39] Sahin S, Eulenburg V, Heinlein A, Villmann C, Pischetsrieder M (2017). Identification of eugenol as the major determinant of GABA_A_-receptor activation by aqueous *Syzygium aromaticum* L. (clove buds) extract. J Funct Foods.

[CR40] Schaefer JE, Zakon HH (1996). Opposing actions of androgen and estrogen on in vitro firing frequency of neuronal oscillators in the electromotor system. J Neurosci.

[CR41] Sîrbulescu RF, Ilieş I, Zupanc GKH (2014). Quantitative analysis reveals dominance of gliogenesis over neurogenesis in an adult brainstem oscillator. Dev Neurobiol.

[CR42] Trowbridge H, Edwall L, Panopoulos P (1982). Effect of zinc oxide-eugenol and calcium hydroxide on intradental nerve activity. J Endod.

[CR43] Wang Z-J, Tabakoff B, Levinson SR, Heinbockel T (2015). Inhibition of Na_v_1.7 channels by methyl eugenol as a mechanism underlying its antinociceptive and anesthetic actions. Acta Pharmacol Sin.

[CR44] Waxman SG, Pappas GD, Bennett MVL (1972). Morphological correlates of functional differentiation of nodes of Ranvier along single fibers in the neurogenic electric organ of the knife fish *Sternarchus*. J Cell Biol.

[CR45] Yalkowsky SH, He Y, Jain P (2010). Handbook of aqueous solubility data.

[CR46] Zupanc GKH (2002). From oscillators to modulators: behavioral and neural control of modulations of the electric organ discharge in the gymnotiform fish, *Apteronotus leptorhynchus*. J Physiol Paris.

[CR47] Zupanc GKH (2020). Development of a sexual dimorphism in a central pattern generator driving a rhythmic behavior: The role of glia-mediated potassium buffering in the pacemaker nucleus of the weakly electric fish *Apteronotus leptorhynchus*. Dev Neurobiol.

[CR48] Zupanc GKH, Maler L (1993). Evoked chirping in the weakly electric fish *Apteronotus leptorhynchus*: a quantitative biophysical analysis. Can J Zool.

[CR49] Zupanc GKH, Banks JR, Engler G, Beason RC, Ploger BJ, Yasukawa K (2003). Temperature dependence of the electric organ discharge in weakly electric fish. Exploring animal behavior in laboratory and field: an hypothesis-testing approach to the development, causation, function, and evolution of animal behavior.

[CR50] Zupanc GKH, Sîrbulescu RF, Nichols A, Ilies I (2006). Electric interactions through chirping behavior in the weakly electric fish, *Apteronotus leptorhynchus*. J Comp Physiol A.

[CR51] Zupanc GKH, Ilieş I, Sîrbulescu RF, Zupanc MM (2014). Large-scale identification of proteins involved in the development of a sexually dimorphic behavior. J Neurophysiol.

[CR52] Zupanc GKH, Amaro SM, Lehotzky D, Zupanc FB, Leung NY (2019). Glia-mediated modulation of extracellular potassium concentration determines the sexually dimorphic output frequency of a model brainstem oscillator. J Theor Biol.

